# Supplementary material for: Gene regulatory network analysis identifies dysregulation of hypoxia pathways as contributing to glioblastoma treatment resistance in females
Source: Biol Sex Differ. 2026 May 21;17:134. doi: 10.1186/s13293-026-00927-4 (PMC13371200; doi:10.1186/s13293-026-00927-4)
Supplement: Supplementary file 1 — Supplementary Material 1. [file 13293_2026_927_MOESM1_ESM.docx]

**S1 Figure. Sex-specific co-regulation obtained using PANDA+BLOBFISH in REMBRANDT** Co-regulation of differentially targeted pathways in female (red) and male (blue) GBMs. “Regulator Count” refers to the number of TFs regulating unique genes in each pathway category, and “Co-Regulator Count” refers to the number of TFs co-regulating unique genes in multiple pathway categories. The filled circles in each column represent the sets of co-regulated pathway categories.
